# Influence of Beam Offset on Dissimilar Laser Welding of Molybdenum to Titanium

**DOI:** 10.3390/ma11101852

**Published:** 2018-09-28

**Authors:** Linjie Zhang, Guangfeng Lu, Jie Ning, Liangliang Zhang, Jian Long, Guifeng Zhang

**Affiliations:** State Key Laboratory of Mechanical Behavior for Materials, Xi’an Jiaotong University, Xi’an 710049, China; ningjie2013@stu.xjtu.edu.cn (J.N.); zhangliangliang1@stu.xjtu.edu.cn (Lia.Z.); longjian528@stu.xjtu.edu.cn (J.L.); gfzhang@mail.xjtu.edu.cn (G.Z.)

**Keywords:** dissimilar joint, laser beam welding, beam offset, pure molybdenum, pure titanium TA2

## Abstract

Dissimilar joining of molybdenum (Mo) to titanium (Ti) is of great significance to the design and fabrication of high-temperature facilities. However, few reports were found about fusion joining of these two metals. The objective of this paper is to assess the feasibility of laser beam welding (LBW) of 2 mm-thick molybdenum and titanium. The effects of laser beam offset on the laser dissimilar joint of pure molybdenum to pure titanium were analyzed in terms of microstructure, chemical composition, microhardness, and tensile behavior. The results showed that the weld appearance improved with the increase of the offset. The fusion zone was strengthened because of the solid solution of these two elements. The mechanical properties of samples increased firstly and then decreased with the increasing of offset. When the laser beam irradiated on the titanium plate and the center of the laser spot was 0.5 mm away from the Mo/Ti interface, the joint performed the highest tensile strength, which was about 70% that of titanium base metal. LBW was demonstrated to be a promising method to join dissimilar Mo/Ti joint.

## 1. Introduction

Molybdenum belongs to the refractory metals and shows good high-temperature mechanical performance and corrosion resistance. It is therefore widely used in fields such as aerospace, electrical industry, chemical industry, and nuclear industry [1]. Titanium, as an important structural material, has high strength, low density, and good corrosion resistance and is widely utilized in industries like aeronautics, chemical, energy, and marine engineering [2,3]. The melting point of molybdenum is about 1000 °C higher than that of titanium, and its coefficient of thermal expansion is about 6 times that of titanium [4]. According to the Ti-Mo diagram [5], the molybdenum shows infinite solubility in β-titanium and limited solubility in α-titanium, and no intermetallics formed in the solid solution of these two metals. In addition, solid solution strengthening effects occurred when titanium and molybdenum mixed with each other [6].

In practice, the dissimilar welding of different metals can reduce structure weight and save production cost. In recent years, there are more and more researches on dissimilar welding of different metals [7,8,9,10,11,12,13,14]. Chen [7] studied the friction stir welding of dissimilar metals, i.e., titanium alloy and aluminum alloy. Through their study, it was found that TiAl_3_ phase appeared on the interface and the maximum tensile strength reached 62% that of the base material (BM) of aluminum alloy. Tomashchuk [8] investigated the electron beam welding of titanium alloy to stainless steel with an interlayer of copper foil. The results showed that when electron beams were concentrated on titanium plates, a large number of brittle phases occurred in the interface. However, when an electron beam was focused on the steel plate, brittle intermetallic compounds were inhibited and the specimens could reach maximum mechanical performance. Ambroziak [4] researched the feasibility of friction welding of molybdenum with other metals, like vanadium, titanium, and tantalum. The results demonstrated that by using friction welding, the dissimilar joints of molybdenum to other metals like vanadium, titanium, and tantalum were successfully achieved and intermetallic phases were not found in welding seam zone. Chang [9] brazed molybdenum with Ti-6Al-4V by using Ti-15Cu-15Ni as the brazing filler metal. A microstructure test indicated that the brazing zone mainly consisted of titanium-rich phases and most brazed specimens fractured in the molybdenum matrix. Du et al. [13] successfully welded the dissimilar joint of 2205DSS to Q235 with laser beam welding. It is found that the welding seam consisted of a martensitic phase and a small amount of residual austenite. However, the HAZ of 2205DSS was mainly comprised of ferrite, and HAZ of Q235 side consisted of a coarse-grained zone and a fine-grained zone.

Although there are a lot of studies on welding of dissimilar metals in recent years, the studies on dissimilar welding of molybdenum and titanium are scarcely carried out. The technical complexity of solid-phase welding and poor fatigue performances of brazing seams limit the applications of these two welding methods in industrial production [15]. And the application of the most widely used welding method in industry, namely the fusion welding, into dissimilar joining Mo/Ti structures, is rarely reported. Laser welding, as a high-energy beam welding method, has numerous advantages, like low heat input and high energy density [16,17,18,19,20,21,22], so it is very suitable for welding refractory metals. Furthermore, there are more and more investigations on laser welding of dissimilar metals in recent years [23,24,25,26]. Zhou [23] studied the dissimilar laser welding of molybdenum and tantalum and found that the generation of cracks on tantalum/molybdenum joints was mainly attributed to the poor weldability of molybdenum. Sun [24] investigated the laser welding of AA6013 aluminum alloys and Q235 low-carbon steels by using ER4043 welding wires. Based on the study, it was found that the thickness of Fe-Al intermetallic compound layers changed with different welding parameters and the specimen fracture occurred in the brazing interface. Song [25] studied the influences of laser offset on Ti6Al4V/A6061 dissimilar welding. The results showed that with the increase of offset, the thickness of intermetallic compound layers decreased and the mechanical performances of the specimens increased. Casalino et al. [26] successfully welded AA5754 and T40 with Yb-YAG laser welding and found that the laser offset significantly affected the ultimate tensile strength of the joints.

This study explored the feasibility of laser beam welding of Mo/Ti dissimilar joints with emphasis on the role of laser beam offsets. The macromorphology and micromorphology of welding seams were observed. By using the energy dispersive X-ray spectrometer (EDX), element distribution in the welding seam zone was tested. The micro Vickers hardness tester and the universal tensile testing machine were used to test microhardness and mechanical performance, respectively. Moreover, the fracture morphology of the specimens was observed by using the scanning electron microscope (SEM).

## 2. Experiment

**Figure 1:**
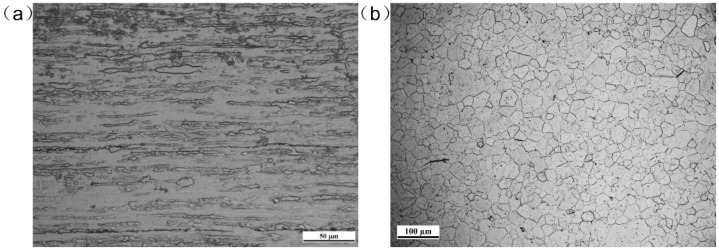
Cross-sectional microstructure of the BMs of (**a**) Molybdenum and (**b**) Titanium.

**Figure 2:**
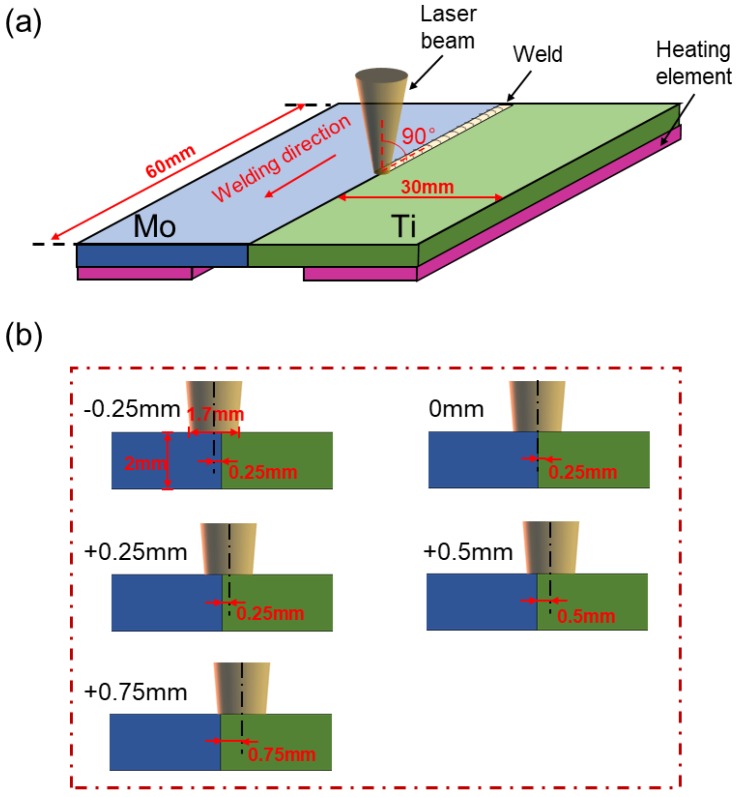
The schematic illustration of (**a**) laser beam offset welding and (**b**) spot size and position at different offsets.

**Figure 3:**
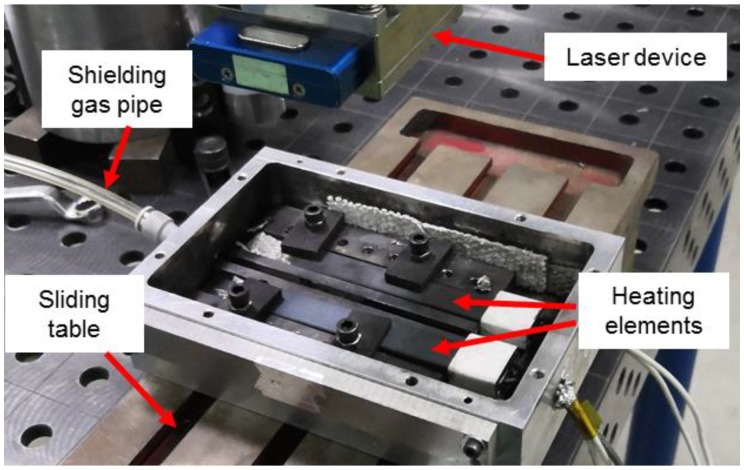
Experimental set-up for argon shielding and preheating.

Pure molybdenum and pure titanium TA2, used in the experiment and the microstructures of the BMs, are shown in Figure 1. The BM of molybdenum was comprised of rolled grains, while the BM of titanium consisted of equiaxed crystals. Five welded joints with different offsets were archived and compared. The welding parameters are shown in Table 1. Laser beam offset indicated the distance between laser spot center on the plates and the Mo/Ti interface. When the center of the laser spot was irradiated on the molybdenum, the offset was recorded as a negative value, while on the titanium plate, the offset was recorded as a positive number. A different offset means different energy distribution on the two plates. In the welding process, molybdenum and titanium plates were preheated to 450 °C and maintained for several seconds with a heating device before welding in argon atmosphere, as shown in Figure 2. After the welding, the heating was stopped and specimens were cooled in the shielding gas. Figure 3 displayed the setting of equipment used in welding test. A sliding table with a regulation precision of 0.01 mm was utilized to change the distance from the laser spot center to Mo/Ti interface and the heating device was placed under the specimens for preheating. Moreover, argon was used as a shielding gas in the welding process. Five different offsets, i.e., −0.25 mm, 0 mm, +0.25 mm, +0.5 mm, and +0.75 mm were employed, as shown in Figure 2b.

The cross-sections of the welded specimens were etched with molybdenum corrodent (i.e., Nitric acid: Sulfuric acid: Water = 5:2:3, by volume, JHD, Guangdong, China) and titanium corrodent (Kroll reagent, JHD, Guangdong, China), successively. The surface morphology of welding seams and micromorphology of cross-sections were observed by using the stereomicroscope (SAIKEDIGITAL, Shenzhen, China) and the metallurgical microscopes (Nikon MA200, Nikon, Tokyo, Japan) separately. The element distribution in the FZ was tested by utilizing the EDX (JEOL, Tokyo, Japan). Moreover, the micro Vickers hardness tester was employed to achieve the distribution of microhardness on cross-sections of specimens. The loading force and duration time were 200 gf and 15 s, respectively. The tensile test was carried out by using the universal tensile testing machine (CSS-88100, SINOMACH, Beijing, China) at a constant drawing speed of 1 mm/min. After the tensile tests, the fracture morphology was observed through the SEM (JEOL, Tokyo, Japan).

## 3. Results and Discussion

### 3.1. Surface Morphologies of Dissimilar Mo/Ti Joints

**Figure 4:**
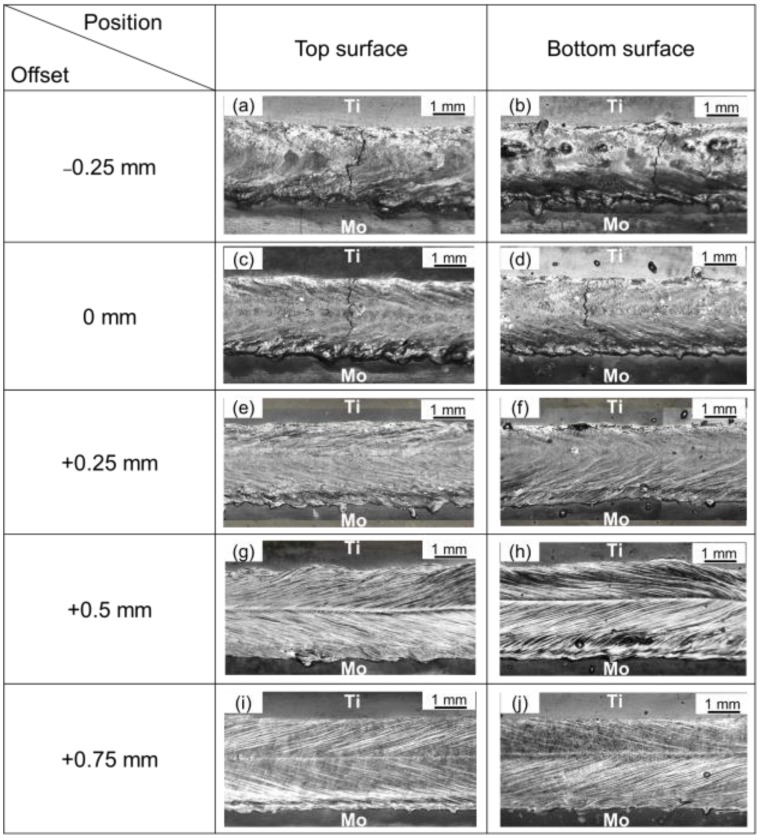
Effects of laser beam offset on the surface morphologies of Mo/Ti joints. (**a**,**c**,**e**,**g**,**i**), the top surface morphologies of welded joints under the offsets of −0.25 mm, 0 mm, +0.25 mm, +0.5 mm, +0.75 mm respectively; (**b**,**d**,**f**,**h**,**j**), the bottom surface morphologies of welded joints under the offsets of −0.25 mm, 0 mm, +0.25 mm, +0.5 mm, +0.75 mm respectively.

Figure 4 shows the top surface morphology and the bottom surface morphology of welding seams achieved under different laser offsets. As demonstrated in this image, laser offset greatly affected the formation of welding seams. When offsets were −0.25 mm and 0 mm, severe transverse cracks were found on both top and bottom surfaces of welding seams and the surface of welding seams was rough and no welding ripples occurred. When the offset was +0.25 mm, there were no obvious cracks on both surfaces of welding seams, but welding ripples were also not obvious. When laser offsets were +0.5 mm and +0.75 mm, a favorable morphology of weld surfaces was formed, showing obvious welding ripples and metallic luster. It can be concluded that with gradually increased laser offset, the formation of welding seams was improved substantially.

### 3.2. Microstructure and Element Distribution

**Figure 5:**
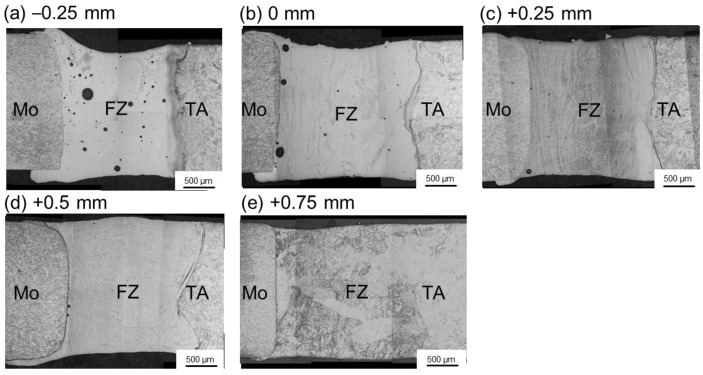
Effects of laser beam offset on the cross-sectional morphologies of Mo/Ti joints. (**a**–**e**), the cross-sections of welded joints under the offsets of −0.25 mm, 0 mm, +0.25 mm, +0.5 mm, +0.75 mm respectively.

**Figure 6:**
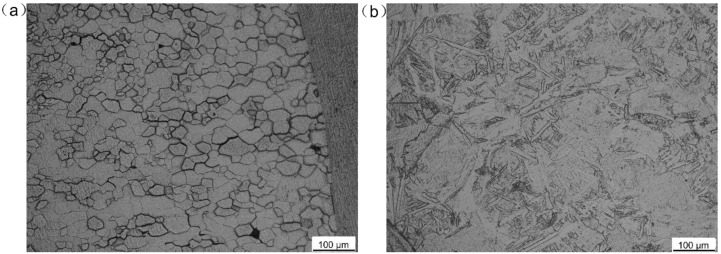
Microstructure of the HAZs (**a**) at molybdenum side and (**b**) at titanium side.

Figure 5 shows the cross-section morphologies of Mo/Ti joints. As displayed in Figure 5a,b, when laser offsets were −0.25 mm and 0 mm, pores appeared in the FZ. When offset was −0.25 mm, plenty of pores appeared and were dispersedly distributed in the whole FZ. While offset was 0 mm, a few pores concentrated around Mo/FZ interface. It is mainly related to the fact that the amount of molybdenum melted in the molten pool scaled with the beam offset. Because of the pre-existing porosity, contamination, and inclusions of molybdenum plate associated to the powder metallurgy process, a higher proportion of molybdenum in the molten pool would result in more porosity defects in the FZ of Mo/Ti joints. Therefore, when laser offsets increased from −0.25 mm to +0.75 mm with an increment value of 0.25 mm, the number of pores decreased monotonically. It can be seen from Figure 5 that the Mo/FZ interface was almost straight, while the FZ/Ti interface was highly curved. Obviously, the straight Mo/FZ interface could be explained by the high melting point and high thermal conductivity of molybdenum. In the welding process, the liquid metal in the molten pool overflowed onto the surface of molybdenum plates and solidified there, as shown in Figure 5. Such a phenomenon gradually weakened with the increase of laser offset. Figure 6a,b displays the HAZs in molybdenum plate and titanium plate, respectively. In the HAZ of molybdenum, recrystallization occurred and equiaxed grains were observed, while martensitic structure was found in the HAZ of titanium.

**Figure 7:**
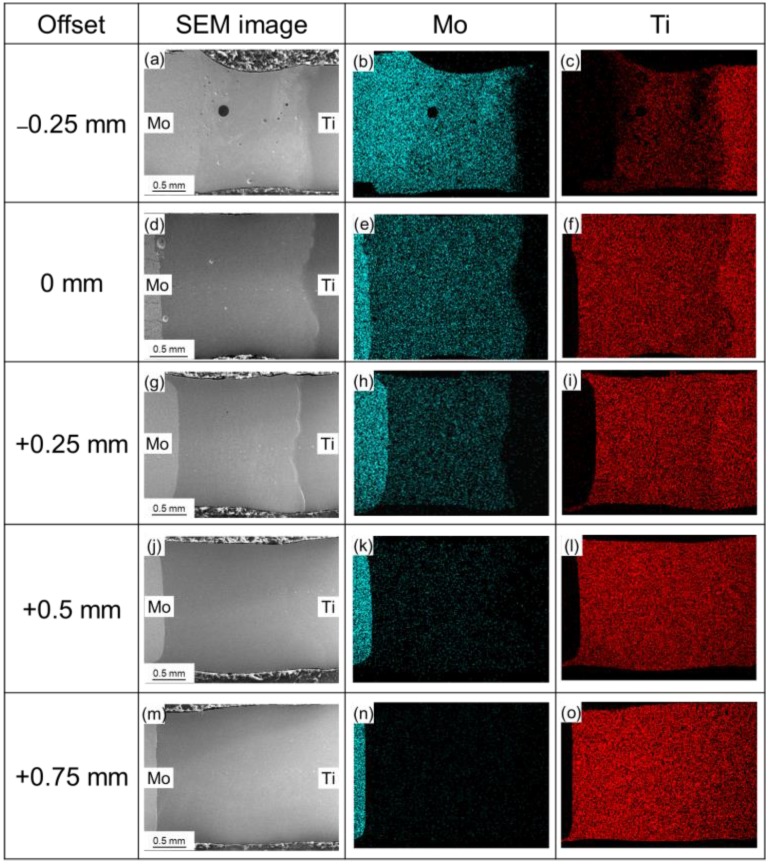
Map scanning results of element distribution for Mo/Ti joints obtained at various laser beam offsets. (**a**,**d**,**g**,**j**,**m**), the SEM image of scanning area of joints −0.25 mm, 0 mm, +0.25 mm, +0.5 mm, +0.75 mm respectively; (**b**,**e**,**h**,**k**,**n**), Mo distribution of joints −0.25 mm, 0 mm, +0.25 mm, +0.5 mm, +0.75 mm respectively; (**c**,**f**,**i**,**l**,**o**), Ti distribution of joints −0.25 mm, 0 mm, +0.25 mm, +0.5 mm, +0.75 mm respectively.

**Figure 8:**
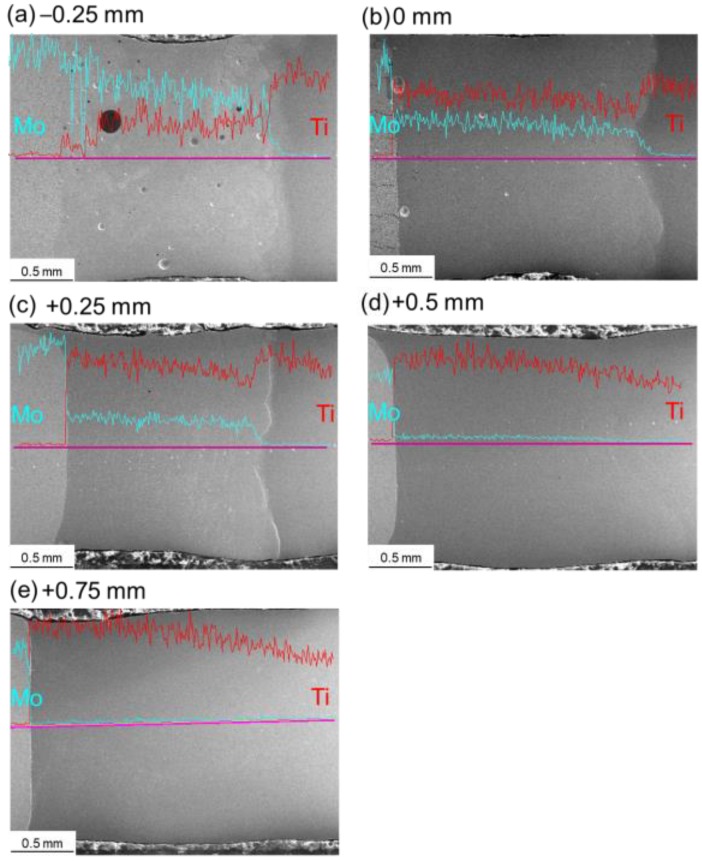
Line scanning results of element distribution for Mo/Ti joints obtained at various laser beam offsets. (**a**) the line scanning result of element distribution of joint −0.25 mm; (**b**) the line scanning result of element distribution of joint 0 mm; (**c**) the line scanning result of element distribution of joint +0.25 mm; (**d**) the line scanning result of element distribution of joint +0.5 mm; (**e**) the line scanning result of element distribution of joint +0.75 mm.

**Figure 9:**
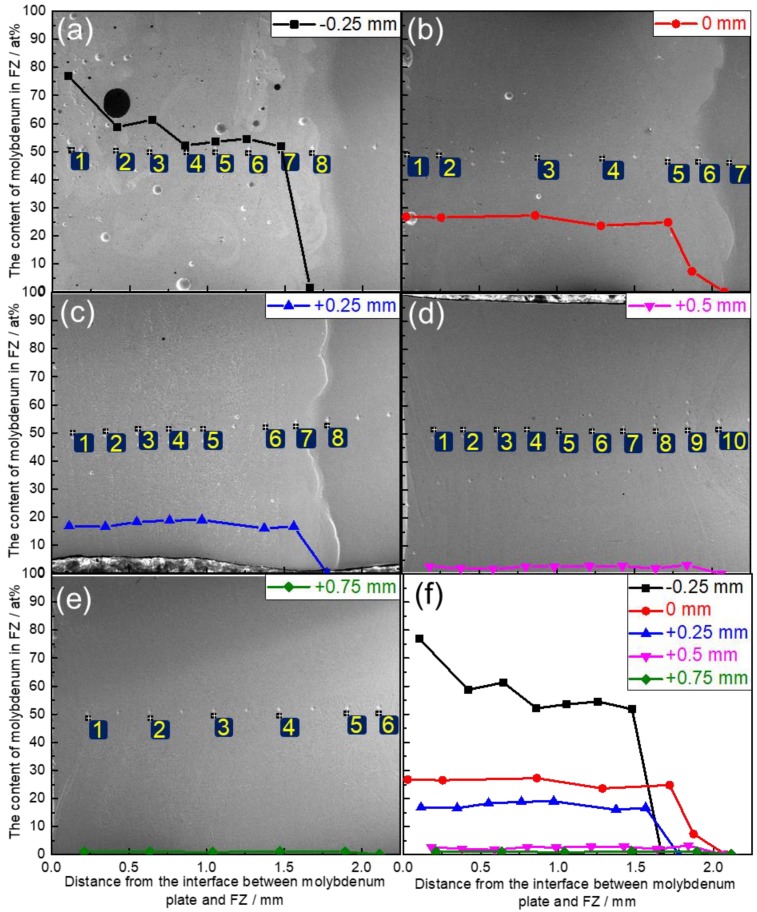
EDX results of the content of molybdenum in Mo/Ti joints obtained at various laser beam offsets (the numbers (1–10) in the blue boxes in this figure represent the positions of point composition analysis). (**a**) point composition analysis result of welded joint −0.25 mm; (**b**) point composition analysis result of welded joint 0 mm; (**c**) point composition analysis result of welded joint +0.25 mm; (**d**) point composition analysis result of welded joint +0.5 mm; (**e**) point composition analysis result of welded joint +0.75 mm; (**f**) the comparison of point composition analysis results of five welded joints.

Figure 7 shows map scanning results of the element distribution on the cross-sections of Mo/Ti joints. It is obvious that both Mo and Ti were uniformly distributed in the FZs after laser beam offset welding. Figure 8 shows the line scanning paths and corresponding elements distribution in the HAZs and FZs of Mo/Ti joints. It can be seen from the figure that for all specimens, the contents of molybdenum and titanium changed gently in the FZ of welding seams, while the contents changed greatly at the FZ/HAZ interfaces. Owing to a higher content of molybdenum in the FZ, when laser offset was −0.25 mm, the content of both elements changed gradually around the Mo/FZ interface. However, for the other four specimens, the contents of both elements changed drastically around the Mo/FZ interface. No titanium was detected in the HAZ of molybdenum. Furthermore, the contents of both elements around the interface between titanium and the FZ changed gently. Figure 9 quantitatively demonstrates the change trend of molybdenum content in the FZ. With the increasing of offset from −0.25 to +0.75 mm by an increment value of 0.25 mm, the contents of molybdenum in the FZs in terms of atomic percent (i.e., Mo_at_) were ranked as: Mo_at_ > 50%, 30% > Mo_at_ > 20%, 20% > Mo_at_ > 15%, 3.5% > Mo_at_ > 1.5%, and 1.5% > Mo_at_ > 0.5%.

### 3.3. Microhardness

**Figure 10:**
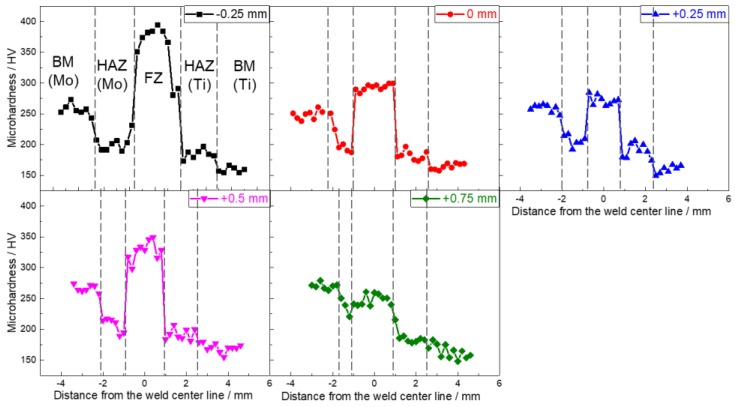
Effects of laser beam offset on microhardness distribution on the cross-sections of Mo/Ti joints.

Figure 10 displays the microhardness distribution along the center line of the thickness direction. Except for the specimens with the offset being +0.75 mm, the FZs of other specimens showed higher microhardness than the BM of molybdenum, which mainly benefited from solid solution strengthening effects [6]. When the offset was −0.25 mm, the microhardness of FZ approaching to the interface between titanium and FZ reduced largely, which was induced by the decrease of content of molybdenum elements at the position (Figure 8a). The microhardness of the HAZ of titanium increased due to the martensitic phase transformation, but owing to the recrystallization, the microhardness of the HAZ of molybdenum declined in comparison with the molybdenum BM.

### 3.4. Tensile Strength and Fracture Observation

**Figure 11:**
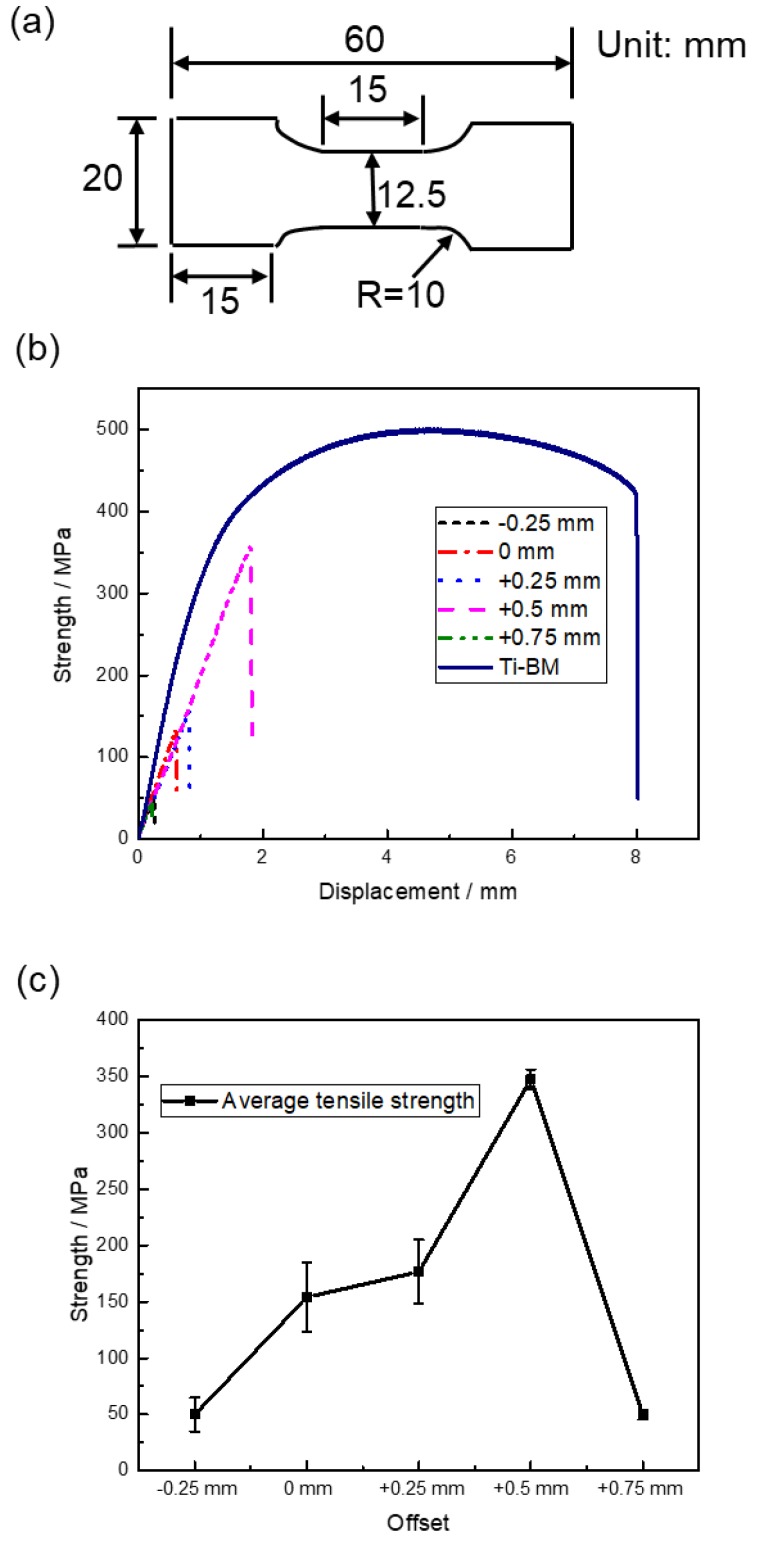
The shape and sizes of tensile specimens (**a**) and results of tensile tests: (**b**) tensile test results of Mo/Ti joints and the BM of titanium, (**c**) variation of average tensile strength of Mo/Ti joint against laser beam offset.

The shape and sizes of tensile specimens and results of tensile tests are shown in Figure 11. Three specimens were tested under each offset and Figure 11b displays the tensile results of one group of specimens. The maximum tensile strength of the specimens was 350 MPa, reaching 70% of the strength of the BM of titanium. Figure 11c shows that with the rise of the offset, the tensile strength of the specimens first increased and then decreased. When offset was +0.5 mm, the specimens showed the largest tensile strength of about 350 MPa. Table 2 shows the yield strength, ultimate strength, and elongation rate of a group of welded joints and BMs. Because of the dimensions of tensile specimens, working easily occurred for the titanium base metal of a welded joint during a tensile test, which caused a higher yield strength and lower elongation rate of joint +0.5 mm than the titanium base metal. All the welded joints showed an unmeasurable elongation rate.

**Figure 12:**
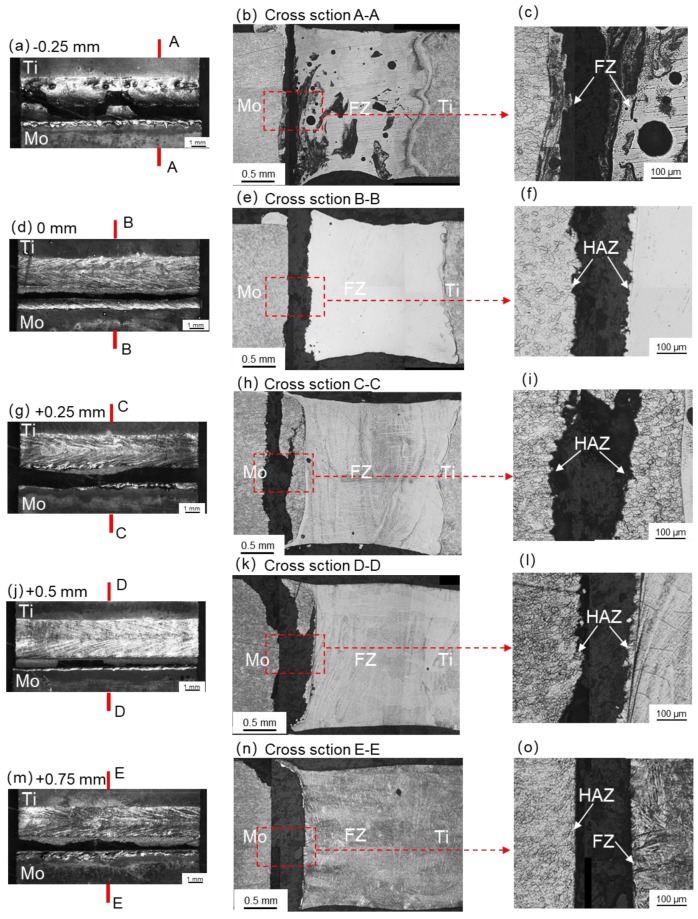
Fracture paths of Mo/Ti joints produced under various laser beam offsets. (**a**,**d**,**g**,**j**,**m**) top surface morphologies of broken joints; (**b**,**e**,**h**,**k**,**n**) cross-sectional morphologies of broken joints; (**c**,**f**,**i**,**l**,**o**) magnified regions in the red boxes in panel **b**, **e**, **h**, **k**, **n** respectively.

Figure 12 shows the tensile fracture path of the welded specimens. When offset was −0.25 mm, due to the presence of defects, such as pores and cracks, the specimen fractured in the FZ, as indicated by the A-A cross-section in Figure 12. When offset was 0 mm, +0.25 mm or +0.5 mm, the specimens fractured in the HAZs of the molybdenum. As a result of the decreasing of its microhardness, the HAZ of molybdenum became the weak area of the joint. When the offset was +0.75 mm, the specimen fractured in the interface between the FZ and the molybdenum plate due to lack of fusion.

**Figure 13:**
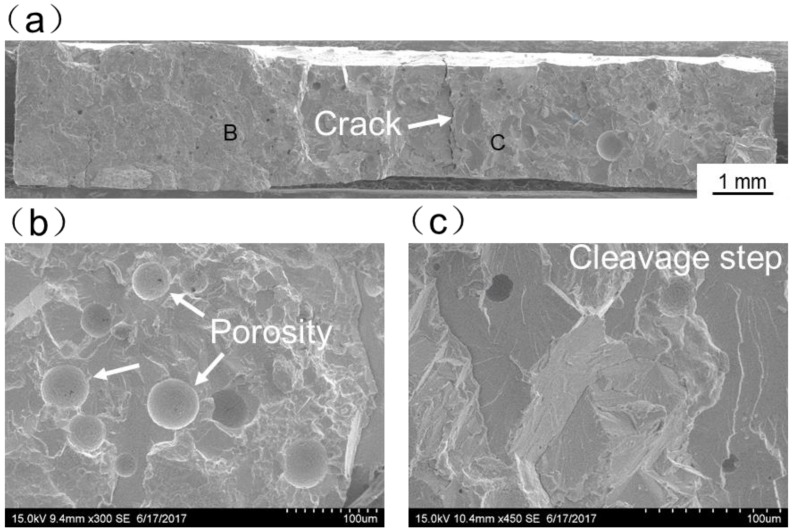
SEM images of tensile fracture when the offset is −0.25 mm ((**a**) Overall morphology; (**b**,**c**) Magnified images of region B and C in panel (**a**)).

**Figure 14:**
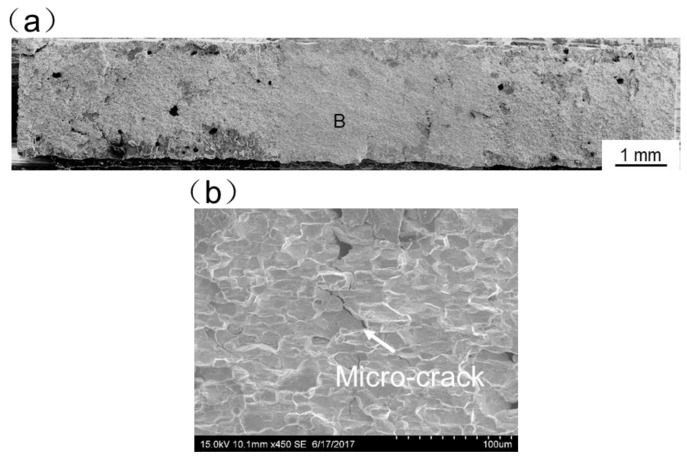
SEM images of tensile fracture when the offset is 0 mm ((**a**) Overall morphology; (**b**) Magnified images of region B in panel (**a**)).

**Figure 15:**
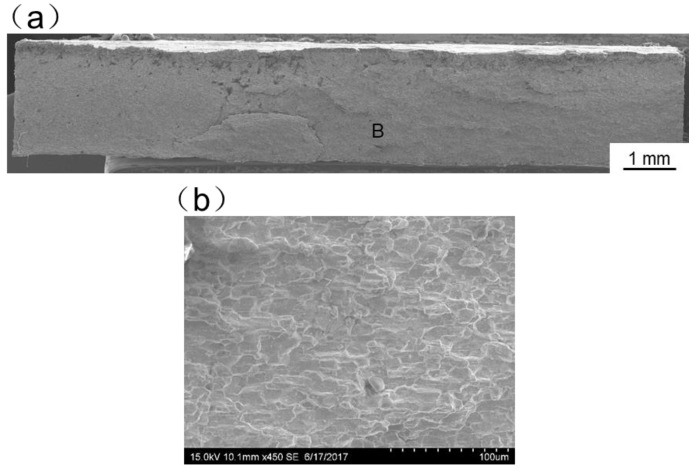
SEM images of tensile fracture when the offset is 0.25 mm ((**a**) Overall morphology; (**b**) Magnified images of region B in panel (**a**)).

**Figure 16:**
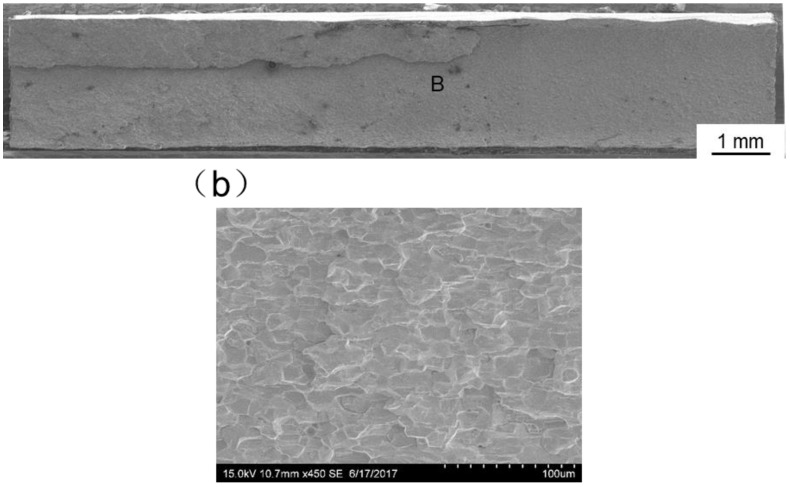
SEM images of tensile fracture when the offset is 0.5 mm ((**a**) Overall morphology; (**b**) Magnified images of region B in panel (**a**)).

**Figure 17:**
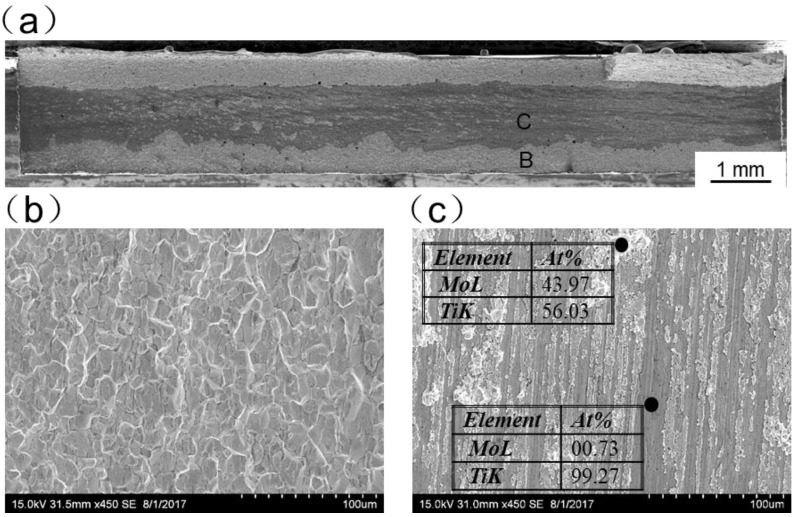
SEM images of tensile fracture when the offset is +0.75mm (**a**) overall morphology; (**b**,**c**) Magnified images of region B and C in panel (**a**).

Figure 13, Figure 14, Figure 15, Figure 16 and Figure 17 display SEM images of tensile fractures of Mo/Ti joints. When offset was −0.25 mm, both pores and cracks running through the thickness direction of the specimens were found on the fracture surface, as can be seen in Figure 13a. The specimen fracture was mainly comprised of cleavage steps. When the offset was 0 mm, micro-cracks were found in the fracture and the fracture mainly presented the characteristics of intergranular fractures, as demonstrated in Figure 14. When the offsets were +0.25 mm or +0.5 mm, the fractures also mainly presented the characteristics of intergranular fractures, as shown in Figure 15 and Figure 16. When offset was +0.75 mm, as shown in Figure 17, the middle part in the thickness direction of the fracture was planar, and its content of titanium was up to about 99.27%, which was similar to the content of the FZ. It is believed that the lack of fusion occurred at the interface of Mo/FZ when the offset was +0.75 mm.

In conclusion, the mechanical performance of the Mo/Ti joints was related to the poor weldability of molybdenum and wetting phenomena of liquid Ti on solid Mo. When offset was small (i.e., −0.25 mm and 0 mm), the pores and cracks in weld seams reduced the mechanical performance. When offsets were +0.25 mm or +0.5 mm, the formation of pores and cracks in the FZ of the specimens was obviously inhibited. Besides at the interface between Mo and FZ, the liquid titanium and solid molybdenum showed relatively better wettability, which can be proved by the fracture positon of these two welded joints. As a result, the strength of specimens increased. In addition, when offset was +0.25 mm, the heat source was much closer to the molybdenum plate, greatly influencing the HAZ of molybdenum. That might result in the lower mechanical performance of specimens produced under the offset of +0.25 mm in comparison with those under the offset of +0.5 mm. However, when offset was too large (i.e., +0.75 mm), at the interface between Mo and FZ, the molybdenum plate was not completely wetted and the lack of fusion occurred, which caused the decrease of strength of welded joints under this laser offset. In addition, around the interface between Mo and FZ, the microstructure and microhardness were quietly different and it caused the very low ductility of welded joints.

## 4. Conclusions

In this work, the influence of laser offset on the weld appearances, alloy element distribution, microhardness, and mechanical properties of laser welded Mo/Ti joints were studied. The main conclusions can be summarized as follows:

(1) Poor weldability of the molybdenum plate, produced by powder metallurgy, greatly affected the mechanical performance of the Mo/Ti joints. Beam offset had a significant influence on the amount of melted molybdenum and therefore plays a crucial role in laser welding of Mo/Ti joints.

(2) When a laser was illuminated on the molybdenum plates, many pores, accompanied with macro-cracks, appeared in the FZ of welded specimens. When laser spots moved towards the titanium plates, macro-cracks disappeared and the formation of pores in the FZs of welded specimens was inhibited.

(3) When laser spots moved from molybdenum plates to titanium plates, the tensile strength of the specimens first increased and then decreased. When offset was +0.5 mm, the specimens showed the maximum tensile strength of about 350 MPa, which was about 70% that of the BM of titanium plate. The HAZ of molybdenum and the interface between molybdenum plate and FZ were the weakest region of Mo/Ti joint.

At last, although it has been demonstrated that the laser beam welding method has the potential to achieve sound Mo/Ti joints, future work to improve the mechanical performance of the joints and the investigation about how the offset affect the wetting behavior of liquid titanium on solid molybdenum is still needed. On the one hand, since that the weakest region of the Mo/Ti joint, which showed the maximum tensile strength of about 350 MPa, was the HAZ of molybdenum, strengthening the HAZ of molybdenum might be an effective way to further improve the strength of laser welded Mo/Ti joint. On the other hand, the mechanism concerning how the offset affects the wetting behavior helps in better understanding the correlation between the laser offset and the strength.

## Figures and Tables

**Table 1:** Parameters used in laser beam offset welding of dissimilar Mo/Ti joint.

Specimen	Power (W)	Welding Speed (m/min)	Defocusing Amount (mm)	Laser Offset (mm)
1	4000	1.5	4	−0.25
2	4000	1.5	4	0
3	4000	1.5	4	+0.25
4	4000	1.5	4	+0.5
5	4000	1.5	4	+0.75

**Table 2:** Tensile result of a group of welded joints and BMs.

Specimen	Yield Stress (Mpa)	Ultimate Stress (Mpa)	Elongation (%)
−0.25 mm	-	59.58	-
0 mm	112.59	156.48	-
+0.25 mm	136.17	189.33	-
+0.5 mm	308.84	346.58	-
+0.75 mm	-	50.22	-
Ti-BM	260.66	500.40	15
Mo-BM	625.55	780.64	4.5
